# Supplementary material for: Resolving the full spectrum of human genome variation using Linked-Reads
Source: Genome Res. 2019 Apr;29(4):635–45. doi: 10.1101/gr.234443.118 (PMC6442396; doi:10.1101/gr.234443.118)
Supplement: Supplemental Material [file supp_gr.234443.118_Supplemental_longranger-2.2.zip › longranger-2.2.tar.bz2!/lr2.2-public/lib/stan/cmdstan/stan/doc/api/html/agrad_2rev_2matrix_2softmax_8hpp_source.html]

Stan: src/stan/agrad/rev/matrix/softmax.hpp Source File


|  |  |
| --- | --- |
|  | Stan  2.5.0  probability, sampling & optimization |


- Main Page
- Related Pages
- Namespaces
- Classes
- Files

- File List
- File Members

All Classes Namespaces Files Functions Variables Typedefs Enumerations Enumerator Friends Macros Pages

- src
- stan
- agrad
- rev
- matrix

softmax.hpp

Go to the documentation of this file.

1 #ifndef STAN\_\_AGRAD\_\_REV\_\_MATRIX\_\_SOFTMAX\_HPP

2 #define STAN\_\_AGRAD\_\_REV\_\_MATRIX\_\_SOFTMAX\_HPP

3

4 #include <vector>

5 #include <stan/math/matrix/Eigen.hpp>

6 #include <stan/math/matrix/softmax.hpp>

7 #include <stan/agrad/rev/var.hpp>

8 #include <stan/math/error\_handling/matrix/check\_nonzero\_size.hpp>

9

10 namespace stan {

11  namespace agrad {

12

13  namespace {

14  class softmax\_elt\_vari : public vari {

15  private:

16  vari\*\* alpha\_;

17  const double\* softmax\_alpha\_;

18  const int size\_; // array sizes

19  const int idx\_; // in in softmax output

20  public:

21  softmax\_elt\_vari(double val,

22  vari\*\* alpha,

23  const double\* softmax\_alpha,

24  int size,

25  int idx)

26  : vari(val),

27  alpha\_(alpha),

28  softmax\_alpha\_(softmax\_alpha),

29  size\_(size),

30  idx\_(idx) {

31  }

32  void chain() {

33  for (int m = 0; m < size\_; ++m) {

34  if (m == idx\_) {

35  alpha\_[m]->adj\_

36  += adj\_ \* softmax\_alpha\_[idx\_] \* (1 - softmax\_alpha\_[m]);

37  } else {

38  alpha\_[m]->adj\_

39  -= adj\_ \* softmax\_alpha\_[idx\_] \* softmax\_alpha\_[m];

40  }

41  }

42  }

43  };

44  }

45

46

57  inline Eigen::Matrix<var,Eigen::Dynamic,1>

58  softmax(const Eigen::Matrix<var,Eigen::Dynamic,1>& alpha) {

59  using Eigen::Matrix;

60  using Eigen::Dynamic;

61

62  stan::math::check\_nonzero\_size("softmax(%1%)",alpha,"alpha",(double\*)0);

63

64  vari\*\* alpha\_vi\_array

65  = (vari\*\*) memalloc\_.alloc(sizeof(vari\*) \* alpha.size());

66  for (int i = 0; i < alpha.size(); ++i)

67  alpha\_vi\_array[i] = alpha(i).vi\_;

68

69  Matrix<double,Dynamic,1> alpha\_d(alpha.size());

70  for (int i = 0; i < alpha\_d.size(); ++i)

71  alpha\_d(i) = alpha(i).val();

72

73  Matrix<double,Dynamic,1> softmax\_alpha\_d

74  = stan::math::softmax(alpha\_d);

75

76  double\* softmax\_alpha\_d\_array

77  = (double\*) memalloc\_.alloc(sizeof(double) \* alpha\_d.size());

78  for (int i = 0; i < alpha\_d.size(); ++i)

79  softmax\_alpha\_d\_array[i] = softmax\_alpha\_d(i);

80

81  Matrix<var,Dynamic,1> softmax\_alpha(alpha.size());

82  for (int k = 0; k < softmax\_alpha.size(); ++k)

83  softmax\_alpha(k) = var(new softmax\_elt\_vari(softmax\_alpha\_d[k],

84  alpha\_vi\_array,

85  softmax\_alpha\_d\_array,

86  alpha.size(),

87  k));

88  return softmax\_alpha;

89  }

90

91

92  }

93 }

94

95 #endif

softmax\_alpha\_

const double \* softmax\_alpha\_

**Definition:** softmax.hpp:17

size\_

const int size\_

**Definition:** softmax.hpp:18

stan::math::softmax

Eigen::Matrix< T, Eigen::Dynamic, 1 > softmax(const Eigen::Matrix< T, Eigen::Dynamic, 1 > &v)

Return the softmax of the specified vector.

**Definition:** softmax.hpp:46

stan::agrad::memalloc\_

memory::stack\_alloc memalloc\_

**Definition:** var\_stack.cpp:16

stan::agrad::vari

The variable implementation base class.

**Definition:** vari.hpp:28

var.hpp

stan::agrad::softmax

Eigen::Matrix< fvar< T >, Eigen::Dynamic, 1 > softmax(const Eigen::Matrix< fvar< T >, Eigen::Dynamic, 1 > &alpha)

**Definition:** softmax.hpp:14

softmax.hpp

Eigen.hpp

stan::memory::stack\_alloc::alloc

void \* alloc(size\_t len)

Return a newly allocated block of memory of the appropriate size managed by the stack allocator...

**Definition:** stack\_alloc.hpp:168

stan::agrad::var

Independent (input) and dependent (output) variables for gradients.

**Definition:** var.hpp:27

stan::math::size

int size(const std::vector< T > &x)

**Definition:** size.hpp:11

alpha\_

vari \*\* alpha\_

**Definition:** softmax.hpp:16

idx\_

const int idx\_

**Definition:** softmax.hpp:19

check\_nonzero\_size.hpp

stan::math::check\_nonzero\_size

bool check\_nonzero\_size(const char \*function, const T\_y &y, const char \*name, T\_result \*result)

Return true if the specified matrix/vector is of non-zero size.

**Definition:** check\_nonzero\_size.hpp:33


---

[ Stan Home Page ]

*© 2011–2014,
Stan Development Team.*
